# Mediators of necroptosis: from cell death to metabolic regulation

**DOI:** 10.1038/s44321-023-00011-z

**Published:** 2024-01-09

**Authors:** Xiaoqin Wu, Laura E Nagy, Jérémie Gautheron

**Affiliations:** 1 Northern Ohio Alcohol Center, Department of Inflammation and Immunity, Cleveland Clinic, Cleveland, OH USA; 2 Department of Integrative Biology and Pharmacology, McGovern Medical School, University of Texas Health Science Center at Houston, Houston, TX USA; 3 Department of Gastroenterology and Hepatology, Cleveland Clinic, Cleveland, OH USA; 4 Department of Molecular Medicine, Case Western Reserve University, Cleveland, OH USA; 5 Sorbonne Université, Inserm UMRS_938, Centre de Recherche Saint-Antoine (CRSA), Paris, 75012 France

**Keywords:** RIP1 Kinase; RIP3 Kinase; MLKL; Cell Death; Metabolism, Autophagy & Cell Death, Metabolism

## Abstract

Necroptosis, a programmed cell death mechanism distinct from apoptosis, has garnered attention for its role in various pathological conditions. While initially recognized for its involvement in cell death, recent research has revealed that key necroptotic mediators, including receptor-interacting protein kinases (RIPKs) and mixed lineage kinase domain-like protein (MLKL), possess additional functions that go beyond inducing cell demise. These functions encompass influencing critical aspects of metabolic regulation, such as energy metabolism, glucose homeostasis, and lipid metabolism. Dysregulated necroptosis has been implicated in metabolic diseases, including obesity, diabetes, metabolic dysfunction-associated steatotic liver disease (MASLD) and alcohol-associated liver disease (ALD), contributing to chronic inflammation and tissue damage. This review provides insight into the multifaceted role of necroptosis, encompassing both cell death and these extra-necroptotic functions, in the context of metabolic diseases. Understanding this intricate interplay is crucial for developing targeted therapeutic strategies in diseases that currently lack effective treatments.

## Abstract (web summary)

This review discusses the different roles of necroptosis, including both cell death and extra-necroptotic functions, in the context of metabolic diseases.

## Graphical abstract

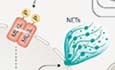

## Introduction

Programmed cell death is a fundamental biological process that regulates cell survival, tissue homeostasis, and various physiological and pathological conditions. Among the different forms of programmed cell death, necroptosis has emerged as a distinct mechanism with unique molecular pathways and functional outcomes. It is characterized by the activation of receptor-interacting protein kinases (RIPKs) and the subsequent phosphorylation and activation of mixed lineage kinase domain-like protein (MLKL), leading to plasma membrane rupture and release of intracellular contents (Dhuriya and Sharma, 2018). While RIPKs and MLKL are critical players in the necroptotic pathway of cell death in pathological settings, emerging evidence suggests that each of the mediators of necroptosis also have extra-necroptotic functions that contribute to the regulation of metabolism, adding complexity to the understanding of necroptosis in the pathophysiology of metabolic diseases (Zhan et al, 2021).

Metabolic diseases, such as obesity, diabetes, alcohol-associated, and non-alcoholic-fatty liver diseases, are complex disorders characterized by dysregulation of metabolic processes. Dysregulated necroptosis has been implicated in the pathogenesis of these metabolic diseases, contributing to chronic inflammation, tissue damage, and altered metabolic homeostasis (Choi et al, 2019). For instance, RIPKs and MLKL have been found to modulate cellular processes involved in energy metabolism, glucose homeostasis, and lipid metabolism. Furthermore, these mediators can influence immune responses, inflammation, and intercellular communication within metabolic tissues (Choi et al, 2019). The interplay between necroptosis-induced cell death and these extra-necroptotic functions highlights the multifaceted role of necroptosis in metabolic regulation.

Understanding the contributions of necroptosis and its extra-necroptotic functions to metabolic diseases is of great importance for developing targeted therapeutic strategies. By unraveling the complex interplay between necroptosis, cell death, and metabolic regulation, we can identify potential therapeutic targets and interventions to mitigate the development and progression of these diseases, which currently lack effective therapeutic options. Therefore, this review aims to comprehensively explore necroptosis and its extra-necroptotic functions in the context of metabolic diseases, shedding light on their intricate relationship and potential implications for therapeutic interventions. We will highlight the current direction for development of small molecule inhibitors in the pathway of necroptosis and their potential for clinical applications. In addition, the controversies and unresolved issues surrounding necroptosis in the field of metabolic regulation, including topics related to cell specific functions and interactions of necroptosis with other cell death pathways, will be discussed, providing a comprehensive overview of the current understanding and highlighting areas for future research.

### Molecular mechanisms of necroptosis

#### Necroptosis signaling pathways

**Figure 1:**
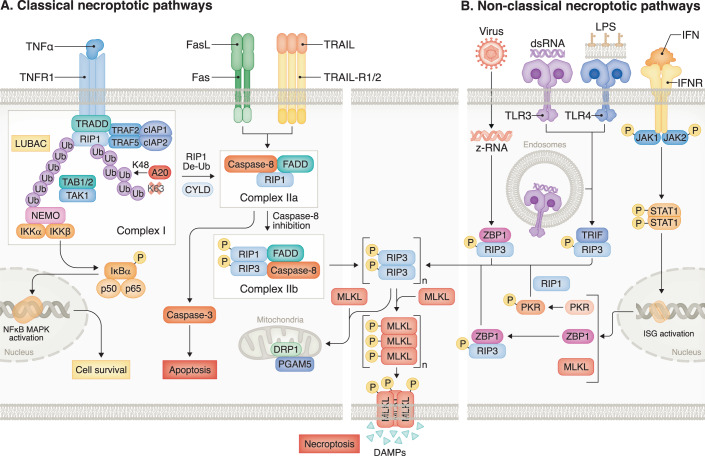
Classical and non-classical necroptotic pathways. (**A**) The classical necroptotic pathways are initiated upon activation of death receptors, including tumor necrosis factor receptor (TNFR) 1, Fas, and TRAIL receptors. This activation subsequently leads to the formation of a membrane signaling complex known as “complex I”, which comprises key components such as TNFR-associated death domain (TRADD), receptor-interacting protein kinase 1 (RIP1), TNF receptor-associated factors 2 and 5 (TRAF2/5), and cellular inhibitor of apoptosis proteins 1 and 2 (cIAP1/2). Complex I serves as a pivotal checkpoint that determines whether the cell will undergo pro-survival or death pathways. TRADD functions as an adaptor molecule that recruits RIP1 to TNFR1. Upon activation, cIAP1/2 and TRAF2/5 mediate the ubiquitination of RIP1, leading to the stabilization of complex I and initiation of alternative pathways, such as NF-κB and MAPK signaling, that ultimately promote cell survival. Once the ubiquitin chain is removed from RIP1, it forms complex IIa through interactions with FADD, TRADD, RIP3, and caspase 8. Under normal conditions, apoptosis is suppressed when caspase-8 forms a heterodimer with cFLIP_L_. Caspase-8, in turn, can induce extrinsic apoptosis. However, when caspase-8 is either eliminated or inhibited, the interaction between RIP1 and RIP3, known as RIP homotypic interactions, then leads to the formation of the complex IIb, initiating the phosphorylation and oligomerization of MLKL, which serves as the executor of necroptosis. (**B**) In addition to the classical pathways, numerous other receptors and intracellular sensors can initiate the non-classical necroptotic signaling cascades. For example, toll-like receptor (TLR)3 and TLR4 activated by double-stranded RNA (dsRNA) and lipopolysaccharide (LPS), respectively, trigger necroptosis directly through a RHIM domain-dependent association of TRIF with RIP3. Viral nucleic acids can activate another RHIM domain-containing protein, ZBP1, which then mediates RIP3-MLKL-dependent necroptosis, independently of RIP1. Moreover, interferon (IFN)-driven necroptosis is mediated via the JAK/STAT-dependent transcription. IFNs can also transcriptionally activate the RNA-responsive protein kinase (PKR), which then phosphorylates RIP1 to trigger necroptosis. Ultimately, all these diverse pathways converge on the activation of MLKL, which is the known executor of necroptosis.

**Overview of the molecular mechanisms of necroptosis and its regulation**. Necroptosis, a type of inflammatory cell death, was first discovered in 2005 (Degterev et al, 2005). Necroptosis primarily relies on the activation of death receptors (DRs), such as tumor necrosis factor receptor (TNFR) 1, Fas/CD95, and TRAIL receptors (Linkermann and Green, 2014) (Fig. 1A). The classical necroptotic pathway triggered by TNF-α involves the binding of TNF-α to its cognate receptor TNFR1, leading to the formation of a membrane signaling complex known as “complex I”. Complex I contains various components, including TRADD, FADD, RIP1 (also referred as to RIPK1), TRAF family proteins, and cellular inhibitor of apoptosis proteins (cIAP) 1/2 and acts as a vital checkpoint that determines whether the cell will undergo pro-survival or death pathways (Weinlich et al, 2017). TRADD serves as an adaptor molecule that recruits RIP1 to TNFR1. Once activated, cIAP1/2 and TRAF2/5 mediate the ubiquitination of RIP1, leading to the stabilization of complex I and initiation of an alternative pathway that ultimately promotes cell survival. This survival pathway includes the activation of NF-кB (nuclear factor kappa B) and MAPK (mitogen-activated protein kinase) signaling. NF-кB signaling plays a critical role in countering the cytotoxic effects of TNF-α, and its pro-survival effects are mediated by cIAP1/2 and cFLIP_L_ (cellular FLICE-like inhibitory protein).

The ubiquitination status of RIP1 determines the balance between pro-survival and cell death pathways. Once the ubiquitin chain is removed from RIP1 through cylindromatosis (CYLD)-mediated deubiquitylation, complex I is destabilized, and RIP1 dissociates from complex I and interacts with FADD through their death domain (Micheau and Tschopp, 2003; Wang et al, 2008). FADD then recruits pro-caspase 8 to form the complex IIa and trigger apoptosis (Majkut et al, 2014). When caspase-8 is inactivated or inhibited, RIP1 can autophosphorylate at S166 and bind with RIP3 through their RIP homology interaction motifs (RHIM) domains, known as RIP homotypic interactions, leading to the formation of the necrosome (complex IIb) and initiating the phosphorylation and oligomerization of MLKL, which serves as the executor of necroptosis. The dynamic process of how endogenous MLKL executes necroptosis was recently identified in human epithelial cells in response to necroptotic stimuli. Basal MLKL is uniformly distributed in the cytoplasm. Upon activation and oligomerization, MLKL coalesces into large cytoplasmic clusters merged with Golgi-derived vesicles of MLKL and then traffics to the plasma membrane along with actin and microtubule filaments and accumulates with tight junction proteins at nascent hotspots until the membranolytic threshold is surpassed, eventually triggering membrane disruption and necroptosis (Samson et al, 2020). MLKL might also act as a platform, either recruiting Na+ or Ca+ + channels (Xia et al, 2016) or promoting pore formation at the plasma membrane through its interaction with the amino terminal of phosphatidylinositol phosphate (Cai et al, 2014). In addition, TNF-α also induces translocation of the RIP1/RIP3/MLKL complex to the mitochondria, where RIPK3 phosphorylates phosphoglycerate mutase family member 5 (PGAM5) at the outer membrane of mitochondria; PGAM5 then dephosphorylates and activates dynamin-related protein 1 (Drp1), subsequently leading to necroptosis (Sun et al, 2012; Wang et al, 2012). However, it is important to note that another study has suggested that the use of shRNA against PGAM5 did not significantly affect necroptosis (Murphy et al, 2013). This may be attributed to incomplete knockdown of PGAM5 or variations in the dependence on PGAM5 in different cell lines, which underscores the complexity of the regulatory mechanisms involved. Overall, the necroptosis machinery is complex, and understanding the role of key components of necroptosis such as RIP1, RIP3 and MLKL, is important to develop the therapeutic strategies targeting this signaling.

**Non-canonical necroptotic pathways**. Multiple plasma membrane-bound receptors and intracellular sensors, in addition to death receptors, can initiate signaling cascades that ultimately lead to the activation of MLKL and MLKL-mediated necroptosis (Fig. 1B). For example, toll-like receptors (TLR3 and TLR4) activated by double-stranded (ds)RNA and LPS, respectively, trigger necroptosis directly through a RHIM domain-dependent association of TRIF with RIP3 (He et al, 2011; Kaiser et al, 2013). TLR4 can also rely on MyD88 for signal transduction (Harberts et al, 2014). Another RHIM domain-containing protein, ZBP1 (also known as DAI or DLM-1), is activated by viral nucleic acids and mediates RIP3- and MLKL-dependent necroptosis independently of RIP1 (Upton et al, 2012). Similarly, interferon (IFN)-driven necroptosis also does not require RIP1 kinase activity; instead, it is mediated via the Jak1/STAT1-dependent transcription by forming a RIP1-RIP3 complex. On the other hand, IFNs can transcriptionally activate the RNA-responsive protein kinase PKR, which binds with RIP1 to induce necrosome formation and trigger necroptosis (Thapa et al, 2013). Therefore, various stimuli induce necroptosis through the activation of multiple receptors, including death receptors, TLRs and IFN receptors, via different pathways involving distinct RHIM-domain-containing protein interactions, including RIP1, RIP3, TRIF and ZBP1. Eventually, all these pathways converge on the activation of MLKL, which is the known executor of necroptosis.

**Post-translational modifications of RIP1, RIP3, and MLKL**. The necroptotic pathway is tightly regulated by multiple post-translational modifications; phosphorylation plays a particularly important role in regulating this pathway. RIPK1 is activated through autophosphorylation at S166, a critical step in regulating RIPK1 kinase-dependent cell death and inflammation (Laurien et al, 2020). However, the death-inducing activity of RIPK1 can be restrained by different kinases. For instance, TAK1 and MAPKAPK2 (MK2) can phosphorylate RIPK1 at S320 (h) or S321 (m) and S336 (m) to limit RIPK1 kinase-induced cell death (Dondelinger et al, 2017; Geng et al, 2017; Jaco et al, 2017; Menon et al, 2017). TAK1 also stimulates IKKα/β, which, in turn, suppresses the death-inducing activity of RIPK1 by phosphorylation at S25 (h&m) (Dondelinger et al, 2019). Furthermore, other kinases like TBK1 and IKKε phosphorylate RIP1 at T189 (h), leading to the inhibition of RIPK1-mediated cell death (Lafont et al, 2018; Taft et al, 2021; Xu et al, 2018). In addition, ULK1, acting as the autophagy-initiating kinase, can restrict the formation of complex IIb by phosphorylating RIP1 at S357 (h) (Wu et al, 2020a).

**Figure 2:**
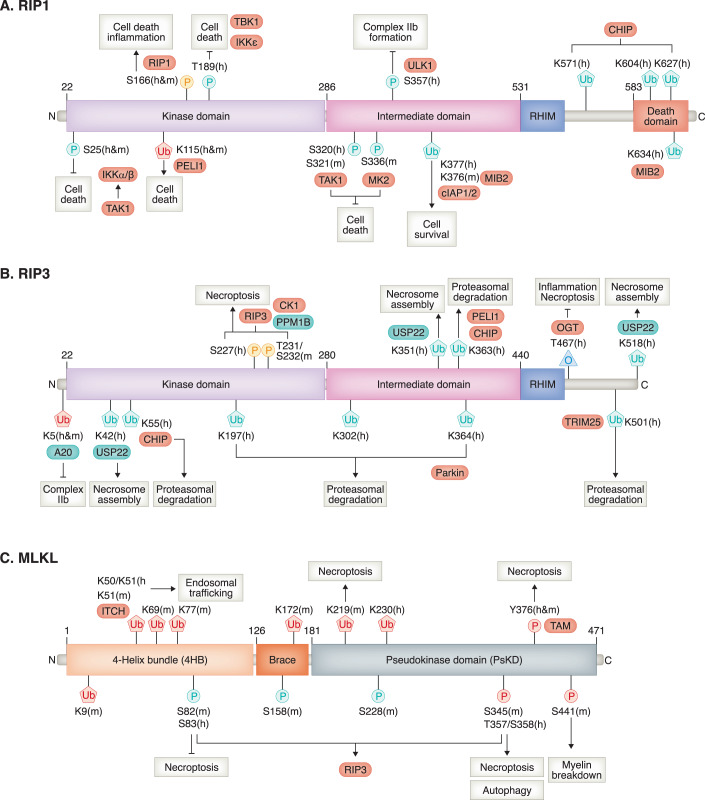
Post-translational regulation of RIP1-RIP3-MLKL-axis and its effect on cell survival and death pathways. Multiple sites on RIP1 (**A**), RIP3 (**B**), and MLKL (**C**) protein have been revealed in the regulation of the balance between cell survival and death signaling by either phosphorylation or ubiquitylation. In addition, human RIP3 is O-GlcNAcylated at T467. P phosphorylation; Ub ubiquitylation. O O-GlcNAcylation. Yellow color of activated site indicates autophosphorylation for activation; Green color indicates inhibition; Red color indicates activation. Upstream molecules for specific sites in circle are also included (Green circle indicates inhibition; red circle indicates activation). Subsequent cellular responses/events are marked in squares.

In addition to phosphorylation, ubiquitination of RIP1 plays a crucial role in TNF-α-mediated cell pro-survival and death signaling (Fig. 2A). Various lysine residues on RIP1 can be ubiquitinated. Pellino 1 (PELI1), an E3 ubiquitin ligase, induced K63-linked ubiquitylation of RIP1 at K115 (h&m), promoting the assembly of death-inducing complexes (Wang et al, 2017). Interestingly, knock-in mutations at K115 did not affect TNF-α-mediated NF-κB and MAPK signaling activation (Kist et al, 2021). However, mutating the cIAPs-targeted ubiquitination site K376 (m) of RIP1 leads to cell death and mouse embryonic lethality (equivalent to K377 in human RIP1) (Kist et al, 2021; Tang et al, 2019). Another E3 ubiquitin ligase, LUBAC, regulates RIP1 linear ubiquitylation (Gerlach et al, 2011; Ikeda et al, 2011; Tokunaga et al, 2011; Witt and Vucic, 2017). Disruption of LUBAC activity exaggerated RIP1 activity and necroptosis (Peltzer et al, 2018; Taraborrelli et al, 2018), suggesting that M1-linked ubiquitylation restricts RIPK1 activity and necroptosis (de Almagro et al, 2017). A20, a potent inhibitor of NF-κB signaling, is also known for stabilizing the ubiquitin scaffold on complex I, thereby counteracting the cytotoxic potential of TNF-α (Priem et al, 2019). There are other E3 ubiquitin ligases implicated in inhibiting necroptosis, such as Mind bomb-2 (MIB2), which ubiquitinates RIP1 at K377 and K634 (Feltham et al, 2018), and carboxy terminus of Hsp70-interacting protein (CHIP, also known as STUB1), which ubiquitinates RIP1 at K571, K604, and K627 (Seo et al, 2016). Further experimental validation is required to fully understand their roles in regulating necroptosis. On the other hand, ubiquitination of RIP1 can be reversed by deubiquitinating enzymes (DUBs). For example, the deubiquitylases CYLD and OTULIN hydrolyze the ubiquitin chains on RIP1, facilitating the formation of complex IIb and promoting necroptosis (Keusekotten et al, 2013; Moquin et al, 2013). Interestingly, A20 also contains separate ubiquitin ligase and DUB domains, which remove K63-linked ubiquitin chains from RIP1 and polyubiquitinate RIP1 with K48-linked ubiquitin chains, thereby promoting the proteasomal degradation of RIP1 (Wertz et al, 2004).

T231/S232 (m) and S227 (h) are sites of auto-phosphorylation on RIPK3 (Chen et al, 2013; Sun et al, 2012). Multiple members of the casein kinase 1 (CK1) family directly phosphorylate RIP3 at these sites (Hanna-Addams et al, 2020; Lee et al, 2019b). One member, CK1G2, employs a different mechanism by physically interacting with RIP3, preventing its homo-dimerization and activation (Li et al, 2020). Protein phosphatase 1B (PPM1B) has also been reported to dephosphorylate RIP3, negatively regulating necroptosis (Chen et al, 2013). Moreover, several E3 ubiquitin ligases, including PELI1 (targeting K363) (Choi et al, 2018), CHIP (targeting K55/K363) (Seo et al, 2016), Parkin (targeting K197/K302/K364) (Lee et al, 2019a), and TRIM25 (targeting K501) (Mei et al, 2021), can induce K48-linked ubiquitylation of RIP3, leading to its proteasomal degradation. In addition, A20 inhibits K63-linked ubiquitination of RIP3 at K5 and disrupts the formation of necroptotic RIP1-RIP3 complexes (Onizawa et al, 2015). In contrast, the ubiquitin-specific peptidase 22 (USP22) removes ubiquitin chains from RIP3 at K42, K351, and K518, promoting necrosome assembly (Roedig et al, 2021). Apart from phosphorylation and ubiquitination, O-GlcNAc transferase (OGT)-mediated O-GlcNAcylation of RIP3 on T467 has been identified to prevent RIP1/3 hetero- and RIP3/3 homo-interaction, thus dampening inflammation and necroptosis (Li et al, 2019) (Schematic illustration in Fig. 2B).

In the classical necroptotic pathway, RIPK3 is crucial in mediating the phosphorylation of MLKL at S345 (m) or T357/S358 (h) (Chen et al, 2013; Sun et al, 2012), leading to a conformational change and oligomerization (Murphy et al, 2013), while in response to serum and amino acid deprivation, calcium/calmodulin-dependent protein kinase II (CAMK2/CaMKII), instead of RIPK3, phosphorylates MLKL on the same sites to facilitate autophagy (Zhan et al, 2022). Interestingly, RIPK3 is also involved in the regulation of inhibitory phosphorylation of MLKL at S82 (m) or S83 (h), S158 (m), and S228 (m) (Tanzer et al, 2015; Zhu et al, 2022). In addition to RIP3, TAM kinases (TYRO3, AXL, and MER) phosphorylate MLKL at Y376 to facilitate its oligomerization (Najafov et al, 2019). In certain cell types, such as Schwann cells, phosphorylated MLKL on S441 contributed to myelin breakdown following nerve injury in mice (Ying et al, 2018). Ubiquitination was also found to play a significant role in regulating the killing capability of MLKL, either promoting or repressing its cytotoxic potential (Fig. 2C). Similar to RIP1 and RIP3, endogenous MLKL can be ubiquitylated on K51, K77, K172, and K219. Among them, K219 was required for MLKL-mediated membrane disruption and necroptotic death (Garcia et al, 2021). Further, another study identified multiple ubiquitylation sites, including K9, K51, K69, and K77 in the 4HB domain. However, by using MLKL-USP21 (a deubiquitylating enzyme which can remove all ubiquitin from MLKL in vitro) fusion, the author found that this fusion can be independent of RIP3 to induce cell death in *Mlkl* deficient cells, suggesting that the ubiquitylation of MLKL also plays a critical role in restraining basal levels of activated MLKL to prevent cell death (Liu et al, 2021a). This might be partially explained by ITCH-mediated poly-ubiquitylation of MLKL on K50/51, which licensed the association of MLKL with endosomes, subsequently resulting in lysosome-mediated degradation or the removal of activated MLKL via extracellular vesicles without triggering necroptosis (Yoon et al, 2022). Together, MLKL can be directed by specific ubiquitylation to differing subcellular sites, thereby it signals either for necroptosis or cell defense mechanism. For example, ubiquitinated MLKL can also fulfill cell death-independent functions, particularly associated with intracellular bacteria clearance (Sai et al, 2019; Yoon et al, 2022).

#### Necroptosis in health and disease

Necroptosis is widely implicated in the pathogenesis of multiple human diseases, including systemic inflammation (Pasparakis and Vandenabeele, 2015), infectious (Xia et al, 2020b), digestive (Dara et al, 2016; Negroni et al, 2020; Wang et al, 2020), respiratory (Sauler et al, 2019), renal (Kolbrink et al, 2023) and cardiovascular diseases (Zhang et al, 2016; Zhe-Wei et al, 2018), genetic diseases (Morgan et al, 2018; Vitner et al, 2014), neurodegenerative and neurological diseases (Daniels et al, 2017; Yuan et al, 2019; Zhang et al, 2017), and cancer (Gong et al, 2019; Najafov et al, 2017; Seehawer et al, 2018). However, whether necroptosis plays a protective or deleterious role in human diseases depends on factors, such as the pathogen species, cell types, and the extent of inflammation and injury. For example, numerous studies have demonstrated that blocking the necroptotic pathway by pharmacological and genetic inhibition yields promising results, while promoting necroptosis in tumors can facilitate tumor suppression (Newton et al, 2016; Zhao et al, 2015). In line with these findings, necroptosis acts as a defensive mechanism against infectious diseases caused by viruses, bacteria and parasites (Cho et al, 2009; Daniels et al, 2017). Some pathogens inhibit necroptosis in host cells through multiple mechanisms; for example, viral inducer of RIP3 degradation (vIRD) leads to K48-mediated RIP3 degradation (Liu et al, 2021b). However, in some cases, such as HIV-1 infection, the virus induces necroptosis in infected CD4+ T lymphocytes and CD4+ T-cell lines, resulting in the progressive loss of CD4+ T cells (Pan et al, 2014). Notably, uncontrolled necroptosis can also lead to excessive inflammation and worsen systemic immune responses and tissue damage during infection. Therefore, the role of necroptosis can be a double-edged sword in the occurrence and development of human diseases.

#### Focus on metabolic diseases

The key mediators of necroptosis are highly involved in various metabolic diseases, such as obesity, diabetes, metabolic dysfunction-associated steatotic liver disease (MASLD; formerly known as NAFLD), metabolic dysfunction-associated steatohepatitis (MASH) and alcohol-associated liver disease (ALD) (Table 1). Studies utilizing RIP1 kinase inhibitors and genetically RIP1 kinase-dead mice have highlighted the significance of RIP1 kinase activity in multiple dietary models of MASLD. For instance, the specific RIP1 inhibitor, RIPA-56, reduced liver steatosis and injury induced by a high-fat diet (HFD) (Majdi et al, 2020). Another recent study demonstrated that genetic inhibition of RIP1 kinase activity (*Rip1*^*K45D/K45D*^) reduced hepatic cell death and inflammation, alleviating hepatic steatosis, liver damage, and fibrosis in both methionine-choline deficient (MCD) and HFD dietary models (Tao et al, 2021). Surprisingly, both kinase-dead *Rip1*^*K45D/K45D*^ and *Rip1*^*S25D/S25D*^ had minimal effect on glucose metabolism and β-cell function in immune-mediated diabetes and diet-induced obesity (DIO) (Takiishi et al, 2023).

**Table 1:** Role of the mediators of necroptosis in metabolic diseases.

Dietary models	Genotypes				
	WT + RIPA-56	*Rip1*^K45D/K45D^	*Rip1*^S25D/S25D^	*Rip3*^*−/−*^	*Mlkl*^*−/−*^
HFD diet	Protective	Protective	No contribution	Deleterious	Partially protective
Western/FFC diet				No contribution	Protective
MCD diet		Protective		Protective	
CD-HFD diet				Protective/no effect	Protective
CDAA diet				Protective	
Gao-binge				Protective	Partially protective
Chronic ethanol				Protective	Partially protective

Interestingly, RIP3, as a substrate of RIP1 kinase, plays diverse roles in various dietary models of fatty liver disease including both ALD and MASLD. *Rip3* deficiency prevented against ethanol- (Roychowdhury et al, 2013; Wang et al, 2016) and MCD diet-induced liver injury (Gautheron et al, 2014), but absence of *Rip3* did not confer protection from either HFD (Roychowdhury et al, 2016) or Western diet-induced (high fat, fructose, and cholesterol, FFC) liver injury (Wu and Nagy, 2020; Wu et al, 2020b). In contrast, in choline-deficient L-amino acid-defined (CDAA) dietary model of MASH, *Rip3* deficiency ameliorates hepatic inflammation and fibrosis and hampered tumorigenesis. Consistent with the mouse data, hepatic RIP3 expression is increased in patients with MASH and correlated with liver inflammation and fibrosis (Afonso et al, 2021). Notably, a previous study observed that *Rip3* knockouts on a choline-deficient (CD)-HFD diet developed more pronounced glucose intolerance, adipose tissue inflammation, and liver injury compared to WT littermates (Gautheron et al, 2016). Conversely, recent work identified that *Rip3* deficiency protected against CD-HFD-induced hepatic inflammation and injury as well as the incidence of hepatocellular carcinoma, particularly in male mice (Mohammed et al, 2023). The conflicting results might be attributed to differences in the pathophysiologic mechanisms in different disease models, variations in genetic backgrounds and/or microbiota across different research facilities.

Recent research has also unveiled distinct roles of MLKL in fatty liver diseases with different etiologies. For instance, studies have reported that *Mlkl*-deficient mice were shielded from liver injury and insulin resistance in murine models of obesity (Mohammed et al, 2023; Wu and Nagy, 2020; Wu et al, 2020b; Xu et al, 2019). On the other hand, *Mlkl* deficiency only partially prevents Gao-binge and chronic ethanol-induced liver injury (Miyata et al, 2021). Further study revealed that cell-specific function of MLKL protected from injury in response to ethanol (Wu et al, 2023). In advanced stage of liver disease, MLKL is strongly associated with the expression of fibrosis-related genes in liver biopsy from patients with cirrhosis. Further study showed that *Mlkl* deletion protected mice from carbon tetrachloride- and bile duct ligation-induced liver injury and fibrosis (Guo et al, 2022). Together, the mediators of necroptosis (RIP1, RIP3, and MLKL) likely function through distinct mechanisms in metabolic diseases. Further investigation is required to fully understand the mechanisms for the regulation of both canonical and non-canonical functions, as well as domain- and cell-specific functions of RIP1-RIP3-MLKL-axis.

Given the significant and distinct roles of the RIP1-RIP3-MLKL axis in metabolic diseases of various etiologies, researchers have investigated the potential of circulating concentrations of RIP1, RIP3, and MLKL as potential biomarkers in humans. Importantly, circulating concentrations of RIP1 and RIP3 distinguished patients with alcohol-associated hepatitis (AH) from healthy controls, as well as from patients with metabolic-associated steatohepatitis (MASH; formerly known as NASH) (Miyata et al, 2021) and RIP3, but not RIP1, is a promising prognostic indicator for patients with AH (Miyata et al, 2021). Circulating RIP3 was also associated with mortality and organ failure during critical illness (Ma et al, 2018). In contrast, MLKL, but not RIP3, was higher in patients with MASH activity score (NAS) ≥ 3 compared to patients with NAS < 3 (Miyata et al, 2021), suggesting circulating MLKL in patients with MASH is reflective of disease severity. Interestingly, another group reported a contrasting finding, revealing a significant increase in serum RIP3 in patients with MASLD (NAS ≥ 2) compared to patients with MASLD (NAS < 2) (Afonso et al, 2021). This discrepancy in results could be attributed to various factors, including differences in sample resources and measurement methodologies. Given the challenges associated with collecting liver biopsy samples, there is a growing need to develop less invasive biomarkers for metabolic liver diseases. Exploring the mediators of necroptosis as a panel of circulating biomarkers holds promise to meet this unmet clinical need. However, to achieve meaningful progress in this area, a standardized and uniform protocol for further research and practical implementation will be essential.

### Non-canonical functions of the necroptosis mediators in metabolism

#### RIP1

RIP1 has recently emerged as a key regulator of the early cell death checkpoint, positioning it as a promising therapeutic target in the context of necroptosis (Ju et al, 2022). Given its pivotal role in cell death regulation, there has been considerable interest in exploring the multifaceted role of RIP1 in the development of metabolic diseases. RIP1 is expressed in the livers of MASH patients and detected in serum of MASH patients with high activity scores (Majdi et al, 2020). In diabetic mice, RIP1 expression is increased in liver, adipose tissue, and muscle, and targeting RIPK1 with a specific inhibitor, necrostatin-1 (Nec-1), prevents insulin resistance and glucose intolerance, independent of its effect on inflammation (Xu et al, 2019). These metabolic effects are likely mediated through the activation of insulin-stimulated AKT signaling, which plays a critical role in glucose homeostasis (Xu et al, 2019).

In the specific context of ALD, studies have investigated the role of RIPK1 and its relationship to necroptosis. Analysis of biopsy samples from ALD and alcohol-cirrhosis patients revealed an increase expression of RIP3 and phosphorylated MLKL, but not RIP1 (Miyata et al, 2022; Zhang et al, 2018). This finding suggests that RIP1 may play a minor role compared to other mediators of necroptosis in these pathological conditions. In addition, the increase in RIP1 expression in the liver was not detected in response to ethanol feeding in mice. In line, inhibiting RIP1 with Nec-1 or 7-Cl-O-Nec-1 did not reduce ethanol-induced hepatocellular damage (Roychowdhury et al, 2013; Wang et al, 2016), in contrast to the effects observed with *Rip3* knockdown (Roychowdhury et al, 2013). These results indicate that ethanol-induced necroptosis is dependent on RIP3 rather than RIP1. Further research is needed to determine whether RIP3 can be activated without the involvement of RIP1 in this specific context.

Studies using *Rip1* kinase-dead (*Rip1*^K45A/K45A^) mice have provided valuable insights into the role of RIP1 in metabolic diseases. These mice exhibited remarkable improvements in MASH phenotype, including reduced hepatic steatosis, liver damage, fibrosis, and lower levels of hepatocellular death and inflammation compared to their wild-type littermates (Karunakaran et al, 2020; Tao et al, 2021). Likewise, a specific inhibitor, RIPA-56, targeting the kinase domain, has demonstrated similar effects (Majdi et al, 2020; Ren et al, 2017). Importantly, RIPK1 inhibition has shown efficacy not only in prophylactic interventions but also in established MASLD, reducing obesity and triglyceride accumulation in the liver (Majdi et al, 2020). Taken together, these data indicate that pharmacological targeting RIPK1 holds promise for the reducing multiple hallmarks of metabolic liver disease including steatosis, inflammation, and fibrosis.

In line with these observations, recent genetic association studies have identified RIP1 as potential target gene for reducing obesity (Karunakaran et al, 2020; Sohrabi and Reinecke, 2021). Inhibition of RIP1 using antisense oligonucleotides (ASOs) has been shown to reduce diet-induced obesity, improve glucose homeostasis, and decrease insulin resistance in mice (Karunakaran et al, 2020), corroborating the beneficial effects observed in mice treated prophylactically with RIPA-56 (Majdi et al, 2020). Interestingly, *Rip1*^K45A/K45A^ mice fed a high-fat diet showed equivalent weight gain to that of WT mice, suggesting that the scaffolding function of RIP1, rather than its kinase activity, regulates obesogenesis (Karunakaran et al, 2020). However, to further understand the specific cellular compartments that contribute to the observed reduction in obesity, the use of conditional knockout mouse models targeting specific cell types, such as adipocytes, hepatocytes, and macrophages, will be essential.

Furthermore, knockdown of RIP1 by ASOs has been found to reduce pro-inflammatory cytokine production in the liver and adipose tissue while promoting the secretion of the anti-inflammatory interleukin (IL)-10 (Karunakaran et al, 2020). The systemic delivery of ASOs suggests that immune cells may play a role in these anti-inflammatory effects. Supporting this idea, chimeric mice transplanted with bone marrow from RIP1 knock out mice and fed a methionine- and choline-deficient (MCD) diet exhibited significantly less fibrosis and steatohepatitis, accompanied by minimal inflammatory markers (Tao et al, 2021).

Recent findings have uncovered the pivotal role of RIPK1 in the intricate interplay between nutrient-sensing pathways and necroptosis (Hardie, 2023; Zhang et al, 2023a). In this context, activated adenosine monophosphate-dependent protein kinase (AMPK) acts as a sentinel for monitoring the nutrient status and energy state of the cell. AMPK phosphorylates and inhibits RIPK1 in nutrient-deprived cells, effectively counteracting necroptosis and suppressing inflammatory responses. However, the duration of nutrient stress can alter this inhibition, eventually allowing cell death to proceed (Hardie, 2023; Zhang et al, 2023a). These discoveries not only emphasize the AMPK-RIPK1 interaction as a critical point of regulation in the balance between cell survival and death but also carry therapeutic implications. The findings provide valuable insights into the interplay of nutrient-sensing pathways with the activity of necroptosis mediators, shaping our understanding of cell fate within the context of metabolic stress.

In conclusion, these findings underscore the multifaceted role of RIP1 in metabolism, inflammation, and cell death regulation, highlighting its potential as a therapeutic target for mitigating metabolic disorders related to obesity. Continued research and exploration of RIP1-targeted interventions holds promise for the development of novel treatments for metabolic diseases. Understanding the precise mechanisms through which RIP1 influences inflammation and metabolic dysfunction will be essential for the development of targeted therapeutics that can effectively modulate these processes and improve metabolic outcome in patients.

#### RIP3

While RIP3 is not expressed in healthy livers (Dara et al, 2015), increased expression of RIP3, an indicator of necroptosis, is detected in livers of patients with MASH (Afonso et al, 2015; Gautheron et al, 2015; Gautheron et al, 2014). Similarly, elevated RIP3 expression has been found in the visceral adipose tissue of individuals with obesity and type 2 diabetes, showing a positive correlation between RIP3 expression, MLKL, and metabolic serum markers (Gautheron et al, 2016). RIP3 has also been found to influence the AKT signaling pathway, further implicating its role in metabolic regulation (Gautheron et al, 2016; Xu et al, 2019). Animal studies have supported these findings, demonstrating increased levels of RIP3 in experimental MASH models (Afonso et al, 2015; Gautheron et al, 2014; Roychowdhury et al, 2016) and following ethanol exposure in both humans with ALD and mice (Miyata et al, 2022; Zhang et al, 2018). Interestingly, *Rip3* deficiency effectively protects against ethanol-induced liver enzyme abnormalities, steatosis, and inflammation (Roychowdhury et al, 2013). However, inhibiting the kinase activity of RIPK1, which normally activate RIP3, only attenuates alcohol-induced hepatic inflammation but does not offer protection against steatosis and liver injury resulting from chronic ethanol consumption (Wang et al, 2016). This suggests that ethanol-induced RIPK3-mediated responses operate independently of RIPK1.

The activation of c-Jun N-terminal kinase (JNK) has been implicated in RIP3-dependent liver damage and fibrosis in mice fed an MCD-diet (Gautheron et al, 2014). Inhibiting JNK reduces RIP3 levels in the liver, suggesting a regulatory role for JNK in RIP3-mediated effects. Interestingly, *Rip3* deficiency protects mice fed a MCD diet from liver injury, inflammation, and fibrosis (Gautheron et al, 2014). However, *Rip3* deficiency exacerbates liver steatosis, apoptosis, adipose tissue inflammation, insulin resistance, and glucose intolerance induced by a HFD (Gautheron et al, 2016; Roychowdhury et al, 2016). Although global knockout of RIP3 in mice does not exhibit any noticeable phenotype (He et al, 2009), the precise mechanism underlying the enhanced apoptosis observed in these mice on an HFD remains uncertain (Gautheron et al, 2016; Roychowdhury et al, 2016). These findings suggest that the effects of *Rip3* deficiency can vary depending on the specific dietary conditions. While it provides protection against liver damage in the context of an MCD diet, *Rip3* deficiency contributes to metabolic disturbances and insulin resistance in the context of a HFD regimen. To gain further insights into this phenomenon, the use of conditional knockout mouse models would allow for the selective targeting of RIP3 in specific cell types, providing a more precise understanding of the complex interplay between RIP3, diet, and metabolic outcomes.

RIP3 has been identified as a regulator of lipid metabolism, exerting differential control over liver steatosis (Zhou et al, 2022). *Rip3* deficiency exacerbates hepatic lipid accumulation induced by a HFD, and this effect is linked to its influence on very low-density lipoprotein (VLDL) secretion markers, including microsomal triglyceride transfer protein (MTTP), protein disulfide isomerase (PDI), and apolipoprotein-B (ApoB) (Saeed et al, 2018). In primary hepatocytes, the absence of RIP3 leads to increased lipid droplets, while its overexpression reduces hepatic lipid accumulation (Saeed et al, 2018). Moreover, *Rip3* deficiency influences the hepatic lipidome in the context of choline-deficient amino acid (CDAA) diet-induced steatohepatitis through the up-regulation of peroxisome proliferator-activated receptor gamma (PPARγ) (Afonso et al, 2021). Functional studies in mice and hepatocytes provide insights into the role of RIP3 in mitochondrial and lipid droplet (LD) biology (Afonso et al, 2023; Islam et al, 2022). RIP3 interacts with key mitochondrial constituents, such as voltage-dependent anion channel (VDAC), mitochondrial antiviral-signaling protein (MAVS), mitochondrial permeability transition pore (mPTP), and metabolic enzymes including pyruvate dehydrogenase (PDH), glycogen phosphorylase (PYGL), glutamate-ammonia ligase (GLUL), and glutamate dehydrogenase 1 (GLUD1) (Islam et al, 2022; Qiu et al, 2018; Yang et al, 2018). These interactions play a role in modulating mitochondrial function and lipid metabolism. The association between RIP3, perilipins (PLIN1 and PLIN5), and disease severity has been demonstrated in patients with MASLD and familial partial lipodystrophy (Afonso et al, 2023). Notably, *Rip3* deficiency improves mitochondrial function and enhances antioxidant systems, reducing stress during lipid overload (Afonso et al, 2023; Conway et al, 1988). Consequently, hepatocytes lacking RIP3 exhibit smaller and increased numbers of LDs (Afonso et al, 2023).

In conclusion, RIP3 has emerged as a key player in liver pathobiology, with its involvement in necroptosis, metabolic regulation, and lipid metabolism. The observed increased expression of RIP3 in conditions like MASH and ALD, as well as its impact on AKT signaling and VLDL secretion markers, highlights its significance in hepatic steatosis and metabolic disturbances. Moreover, the interaction between RIP3 and mitochondrial constituents and its influence on the hepatic lipidome further emphasize its role in lipid metabolism. Future exploration using conditional knockout mouse models will be crucial in deepening our understanding of the complex interplay of RIP3 in hepatic steatosis. Since data indicate that RIP3 can be activated independently of RIP1, development of specific pharmacological inhibitors of RIP3 will likely be necessary in some conditions to interrupt the pathophysiological impact of RIP3 in progression of liver diseases.

#### MLKL

MLKL is induced and activated by different high-fat diets in murine models of metabolic disease (Saeed et al, 2019; Wu et al, 2020b; Xu et al, 2019). Similarly, the induction and phosphorylation of MLKL are observed in cell culture models in response to stimuli, such as free fatty acid (Wu et al, 2020b) or LPS (Wu et al, 2023). In patients with MASLD and obesity, MLKL is also highly expressed and phosphorylated in multiple organs including liver and adipose tissue (Magusto et al, 2022; Wu et al, 2023) as well as multiple cell types, including liver parenchymal and non-parenchymal cells, such as hepatic stellate cells and immune cells (Guo et al, 2022; Wu et al, 2023). Nonetheless, how MLKL expression is regulated in different cells and tissues at the transcriptional level remains poorly understood. One notable finding is that STAT1-dependent signaling can regulate the transcription of MLKL as an IFN-stimulated gene (ISG) in IFN-γ-treated hepatocytes (Gunther et al, 2016). STAT1 is also involved in LPS-mediated induction of MLKL in hepatic macrophages (Wu et al, 2023). In other cell types, such as endothelial and smooth muscle cells, long non-coding RNA (lncRNA)-FA2H-2 was found to interact with the *Mlkl* promoter, downregulating its expression during atherogenesis (Guo et al, 2019). Thus, the mechanisms governing the interplay between obesity/high-fat diet and MLKL induction may vary depending on the cell type and specific stimulus.

In the past decade, accumulating evidence has revealed MLKL functions beyond mediating necroptosis. Baseline expression of MLKL in cells, such as Kupffer cells, is unexpectedly essential for maintaining immune cell function and liver homeostasis (Wu et al, 2023). Importantly, studies have highlighted the pivotal role of MLKL-mediated non-canonical functions in the pathogenesis of MASLD and obesity. Protection of *Mlkl*-deficient mice from different types of high-fat diets is likely associated with the regulation of lipid-associated metabolic processes, including lipogenesis and cholesterol biosynthesis (Saeed et al, 2019). MLKL-dependent signaling can modulate the expression of molecules involved in lipid uptake, transport, and metabolism, thereby influencing the hepatic lipidome (Saeed et al, 2019), (10.1101/2023.03.14.532682, not accepted yet). These non-canonical roles of MLKL in lipid metabolism may be related to MLKL localization within nuclei and its ability to regulate the expression of lipid-related genes, particularly in the context of high-fat diets. Another nuclear function of MLKL is in response to LPS or TNF-α stimulation, where MLKL translocates to the nucleus and governs mRNA expression of genes involved in phagocytic and adhesion activities (Dai et al, 2020; Wu et al, 2023). Further evidence indicates that activated MLKL co-localizes to the nuclear membrane and sites of DNA release at the plasma membrane in neutrophils, facilitating nuclear membrane breakdown and chromatin decondensation, ultimately leading to the formation of neutrophil extracellular trap (D’Cruz et al, 2018).

**Figure 3:**
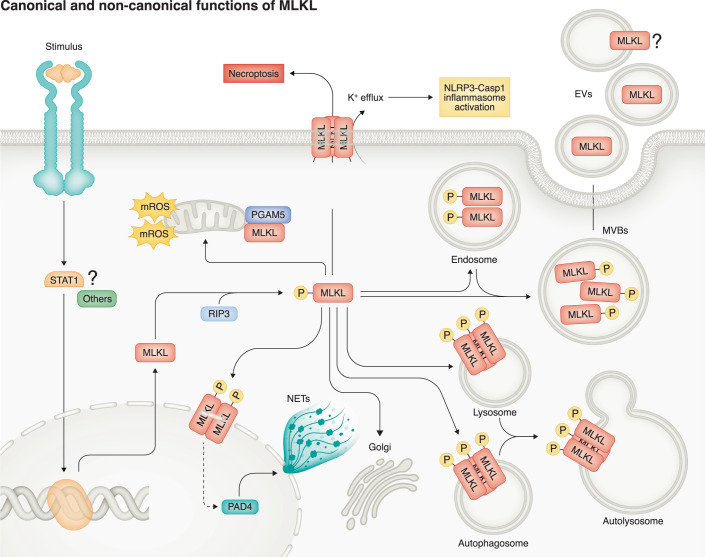
Subcellular localization and canonical and non-canonical functions of MLKL. MLKL is induced and activated in different cells and tissues in response to various stimuli. However, the underlying mechanisms governing the induction of MLKL remain poorly understood. Recent studies have found that STAT1-dependent signaling can regulate the transcriptional expression of MLKL as an IFN-stimulated gene in hepatocytes as well as LPS-treated macrophages. Activated MLKL, through RIP3 or alternative mechanisms, can translocate to multiple intracellular compartments, including mitochondria, endosomes, and Golgi. Furthermore, MLKL exhibits the ability to move into multivesicular bodies (MVBs), subsequently undergoing exclusion from cells via extracellular vesicles (EVs). In addition, the translocation of MLKL to either autophagosomes or lysosomes serves to inhibit their fusion, thereby suppressing the formation of autolysosomes. Compelling evidence further underscores the nuclear translocation of MLKL and its role in regulating gene expression. Notably, MLKL facilitates nuclear membrane breakdown and chromatin decondensation, ultimately resulting in the formation of neutrophil extracellular traps (NETs).

Important for understanding the complex functions of MLKL, it is now clear that MLKL exhibits multiple novel non-canonical functions in addition to its translocation to and disruption of the plasma membrane. Activated MLKL has the ability to translocate to multiple intracellular compartments, including mitochondria (Deragon et al, 2023; Wang et al, 2014), endosomes (Wu et al, 2023; Yoon et al, 2022; Yoon et al, 2017), lysosomes (Liu et al, 2018; Wu et al, 2023), and Golgi (Wu et al, 2023). MLKL can also translocate to multivesicular bodies (MVBs) and be subsequently excluded from cells through extracellular vesicles (Yoon et al, 2017). These non-canonical functions of MLKL are important in metabolic liver diseases. For example, translocation of MLKL to autophagosomes in response to FFC diet or palmitic acid serves to inhibit the fusion of autophagosomes with lysosomes, thereby suppressing the degradation of injurious intracellular components, including lipid droplets, in hepatocytes. Since autophagy serves as a protective mechanism for breaking down lipid droplets, the process of MLKL-mediated biogenesis and breakdown of lipid droplets likely plays a vital role in the initiation and development of MASLD (Wu and Nagy, 2020; Wu et al, 2020b). In summary, these findings underscore the versatile and dynamic functions of MLKL in multiple cellular processes, contingent on its specific subcellular localization (Fig. 3). However, it is crucial to note that some studies come with inherent limitations, as they rely on fractionation experiments that may not directly demonstrate subcellular localization and function (Deragon et al, 2023). Furthermore, questions arise regarding the use of GFP-MLKL fusions in experiments and how accurately they represent the behavior of endogenous MLKL (Yoon et al, 2017). Robust evidence supports MLKL’s primary role in plasma membrane translocation during necroptosis induction, as demonstrated in high-quality studies, such as the findings of Sansom et al (Samson et al, 2020). Nevertheless, the absence of a direct one-to-one relationship between MLKL translocation and plasma membrane disruption raises intriguing questions. This suggests the involvement of additional factors or interactions that influence MLKL’s diverse intracellular behavior. In essence, while the translocation of MLKL to the plasma membrane is undeniably significant, it does not provide a comprehensive explanation for its multifaceted roles within the cell. This implies the likelihood of other mechanisms or interactions that contribute to its functions in various cellular compartments.

In addition to its protective effects on hepatic lipotoxicity, macrophage MLKL is required for phagocytic capability in response to ethanol/LPS challenge (Wu et al, 2023). Further, MLKL contributes to HSC activation during differentiation to activated myofibroblasts in fibrosis (Guo et al, 2022; Pistorio et al, 2022). Similarly, the absence of *Mlkl* suppresses differentiation of white adipocytes and secretion of adipokines and cytokines (Magusto et al, 2022). This effect may be associated with the downregulation of Wnt10b, a ligand of the Wnt/β-catenin pathway, leading to decreased expression of genes involved in lipid metabolism (Magusto et al, 2022). Considering that obesity/MASLD is a systemic disease, it would also be intriguing to assess how MLKL functions in other organs, such as the gut and adipose tissue. Exploring MLKL’s actions in these tissues and cells and how MLKL mediates cell-to-cell interaction could provide valuable insights into its broader impact on disease development and progression.

Taken together, MLKL emerges as a strong candidate highly implicated in the regulation of balance between cell death and pro-survival pathways, including autophagy and vesicle trafficking. However, several crucial questions remain unanswered, warranting further investigation to gain deeper insights into the mechanisms through which MLKL is involved in this dysregulation within specific cells in diverse murine injury models. The interaction of MLKL with multiple intracellular vesicles in different cell types and tissues remains understudied. For example, it is not known whether these non-canonical functions of MLKL can influence homeostasis and disease progress independently on RIPK3-mediated activation of MLKL and MLKL-driven necroptosis. Neglecting these functions could potentially lead to serious side effects when MLKL inhibitors are employed for therapeutic purposes. Moreover, while MLKL oligomers have been observed in intracellular membrane fractions, it remains unclear whether these non-classical functions of MLKL require its oligomerization and pore formation at the membrane. Further, little is known how MLKL-dependent signaling is mechanistically related to the same biochemical pathways underlying other cell death modalities, including pyroptosis and ferroptosis. Therefore, there is an ongoing and profound interest in conducting further investigations to unveil additional mechanisms involved in the activation and function of MLKL. This will contribute to a more comprehensive understanding of its role in cell fate regulation and potentially reveal new therapeutic opportunities in various disease contexts.

More importantly, elucidating the role of MLKL in human disease in the context of diverse genetic backgrounds and environmental challenges remains limited. From a clinical perspective, only a limited number of human diseases, including metabolic disorders, have been associated with variant sequences of MLKL to date. One recent study identified a rare mono-allelic damaging mutation in the MLKL gene exclusively present in diabetic family members (Hildebrand et al, 2021). While these cases are rare, they hold the potential to provide valuable insights into the role of MLKL in disease etiology. They may contribute to a more focused and refined study of the trait- and disease-modifying potential of MLKL protein-coding variants carried by over 10% of the global population (Garnish and Hildebrand, 2022), leading to the identification of new clinical indications and contraindications for drugs targeting necroptosis and its upstream regulators. Such investigations will shed further light on whether the absence of MLKL alone is sufficient to affect metabolic disease or if other genetic or environmental factors are also involved in disease development. Understanding these complex interactions will be crucial in unraveling the precise impact of MLKL on human health and disease in real-world scenarios.

### Controversies, unsolved issues and recommendations

#### The differential expression of necroptosis mediators in hepatocytes and adipocytes: insights into cell-specific variations

While MLKL and RIP1 are widely expressed throughout the body, the expression of RIP3 varies among different cell types. Notably, RIP3 is highly abundant in immune cells (Lu and Walsh, 2012), keratinocytes (Dannappel et al, 2014), and intestinal epithelial cells (Dannappel et al, 2014; Takahashi et al, 2014). However, its basal expression in hepatocytes appears to be absent (Dara et al, 2015; Van et al, 2017). This raises an intriguing question regarding the susceptibility of hepatocytes to undergo necroptosis. Necroptosis occurs when caspases are either absent or their activity is inhibited. Complete knockout of caspase-8 leads to lethal necroptosis, which can be prevented by removing either RIP3 or MLKL (Tummers and Green, 2017). Surprisingly, hepatocyte-specific knockout mice lacking caspase-8 using *alb*-cre show no noticeable changes (Kaufmann et al, 2009; Postic et al, 1999), but when caspase-8 is specifically deleted using the *alfp*-cre line, a distinct phenotype characterized by the presence of necrotic areas, elevated levels of transaminases, and bilirubin in the blood, is observed (Gautheron et al, 2014; Kellendonk et al, 2000; Liedtke et al, 2011). This disparity may be attributed to the *alfp*-cre line’s ability to delete the floxed alleles in all liver parenchymal cells, including cholangiocytes and progenitor cells, which might exhibit a higher susceptibility to necroptosis under normal conditions. Moreover, the release of inflammatory mediators by these susceptible cells could potentially trigger further cell death events, such as apoptosis, in hepatocytes.

Similarly, under basal conditions, RIP3 does not appear to be expressed in adipocytes (Gautheron et al, 2016; Magusto et al, 2022). However, in pathological conditions, the expression of RIP3 is dramatically increased in the liver and adipose tissue (Khoury et al, 2020). Determining whether this upregulation primarily occurs intrinsically in hepatocytes and adipocytes or is a result of infiltrating inflammatory cells remains challenging. The inflammation generated by these immune cells may play a crucial role in the pathogenesis of metabolic diseases, triggering programmed cell death events independent of necroptosis in adipocytes and hepatocytes. These observations highlight the critical importance of maintaining a delicate balance in the regulation of RIP3 expression. Insufficient RIP3 expression can compromise cellular defense responses, while excessive levels can lead to increased susceptibility to necroptosis, which can be detrimental to the organism (Morgan and Kim, 2022).

Recent studies have shed light on the regulation of RIP3 expression, revealing that it is modulated by hypermethylation of its promoter region (Hoff et al, 2023; Preston et al, 2022). Interestingly, this hypermethylation was found specifically in hepatocytes but not in RAW macrophages (Preston et al, 2022), and may be likely to also occur in adipocytes which do not express RIP3 basally. This suggests that the effects observed in pathological conditions are likely associated with infiltrating immune cells. However, since DNA methylation/demethylation processes are dynamic, it is possible that under pathological conditions, demethylation of the CpG islands in the RIP3 promoter occurs, enabling the initiation of necroptosis when apoptosis is inhibited. Nevertheless, formal demonstration of this hypothesis is still required. This scenario bears resemblance to what was observed in a clinical trial involving the use of a pan-caspase inhibitor, emricasan (Harrison et al, 2020). The trial was discontinued due to adverse effects in patients with MASH, including elevated transaminase levels (Harrison et al, 2020). These findings raise the question of whether the promoter demethylation of RIP3 in hepatocytes could have occurred in the context of lipid overload or immune cells undergoing necroptosis, subsequently triggering a cascade of events.

In conclusion, a thorough investigation of the interactions between parenchymal and non-parenchymal cells is essential to unravel the mechanisms underlying cell death events in the liver and adipose tissue. The conflicting findings regarding RIP3 expression and regulatory mechanisms highlight the necessity for the development of specific conditional knockout models. These models will provide a more targeted approach to studying RIP3 and its role in cell death pathways. Such knowledge is crucial for identifying potential therapeutic targets and designing interventions for liver and metabolic diseases.

#### From cytoplasm to nucleus: are necroptosis mediators key regulators of gene expression?

In addition to their cytoplasmic functions, mediators of necroptosis have been shown to localize to the nucleus, suggesting their potential role in the regulation of gene expression. One notable example is MLKL, which translocates to the nucleus prior to the execution of necroptosis, independent of its association with the nuclear membrane (Yoon et al, 2016). Interestingly, MLKL’s nuclear localization is not essential for its lethal function, as inhibiting necroptosis with necrosulfonamide (NSA) does not affect this localization (Yoon et al, 2016). Despite lacking DNA or RNA binding domains, MLKL has been implicated in indirect transcriptional regulation by interacting with factors that possess these domains (Zhan et al, 2021). Supporting this notion, recent research has demonstrated the interaction between MLKL and RNA-binding motif protein 6 (RBM6), leading to enhanced expression of adhesion molecules in endothelial cells (Dai et al, 2020).

Similarly, RIP1 has been implicated in transcriptional regulation by directly binding to BRG1/BRM-associated factor (BAF) chromatin-remodeling complex proteins, SMARCC2 and BRG1 (Dai et al, 2020). This interaction triggers the phosphorylation of SMARCC2, resulting in chromatin remodeling and transcription of pro-inflammatory genes (Dai et al, 2020; Gullett and Kanneganti, 2022). In addition, nuclear RIPK1 undergoes ubiquitination upon stimulation with TNF and is suggested to interact with nuclear RIPK3 for its activation (Weber et al, 2018). When NLRP3 inflammasomes are induced, all necroptosis mediators localize to the nucleus (Weber et al, 2018). These findings reinforce the notion that necroptosis mediators have the ability to exert control over gene expression and underscore their multifaced role in cellular processes. In the context of metabolic diseases, it is crucial to consider the potential involvement of necroptosis mediators in transcriptionally regulating genes involved in metabolism, as suggested by studies conducted in liver (Afonso et al, 2021) and adipocytes (Magusto et al, 2022). Further investigations in these areas could provide valuable insights into the pathogenesis of metabolic disorders and identify potential therapeutic targets.

#### Interplay of cell death modes: PANoptosome-mediated regulation of necroptosis, pyroptosis and apoptosis

Recent evidence points towards the involvement of necroptosis mediators in a lethal protein complex called the PANoptosome (Samir et al, 2020). This complex orchestrates a synchronized response to cell death, encompassing pyroptosis, apoptosis, and necroptosis, collectively referred to as PANoptosis. Functioning as a convergence point, the PANoptosome allows the activation of one pathway to influence the activation of others. For instance, both RIPK1 and RIPK3 can be cleaved by caspase-8 (Feng et al, 2007; Newton et al, 2019), modulating their activation. Intriguingly, mice lacking *Rip3* or carrying mutations in its kinase domain display increased susceptibility to apoptosis, either in pathological conditions or spontaneously, highlighting the interconnected nature of various cell death modes (Gautheron et al, 2016; Mandal et al, 2014b; Roychowdhury et al, 2016).

Similarly, RIP1, initially recognized as a modulator of NF-кB signaling through its interaction with IKKs (Kondylis et al, 2017), is now acknowledged as a critical early checkpoint in determining cell fate (Newton, 2020). RIP1 not only controls NF-кB activation, promoting cell survival, but also acts as a key regulator of RIP3, influencing both necroptosis and apoptosis (Newton, 2020). In addition, RIP1 interacts with poly (ADP-ribose) polymerase (PARP) and has been implicated in peroxide-induces PARP-1-mediated necrosis (Jang et al, 2018). Post-translational modifications of RIP1, including ubiquitination and phosphorylation, further modulate its function, adding an extra layer of complexity to the regulation of cell death (Varfolomeev and Vucic, 2022).

MLKL is not left behind and has been implicated in the regulation of pyroptosis as well (Conos et al, 2017). Pyroptosis is a lytic form of cell death triggered by nucleotide-binding oligomerization domain (NOD)-like receptors and the NLRP3 inflammasome, along with the cleavage of Gasdermin D (GSDMD) (Yu et al, 2021). Remarkably, MLKL can activate the NLRP3-caspase-1 inflammasome and induce the secretion of IL-1β as a result of necroptosis signaling, occurring prior to cell lysis (Conos et al, 2017). Importantly, in this context, the pore forming substrate GSDMD, which is cleaved by caspase-1, is not required for IL-1β release (Conos et al, 2017). These findings suggest that different forms of regulated cell death can coexist in certain cases, making it challenging to clearly define the predominant type of cell death in specific pathological conditions. While inhibiting all effectors of cell death pathways simultaneously may not be feasible, a more viable approach could involve targeting one or more mediators of necroptosis that contribute significantly to the convergence of cell death pathways. Such a targeted approach may offer a more effective strategy for treating metabolic diseases involving dysregulated cell death.

#### Improving preclinical models and rigor for MASH/ALD research and metabolic disorders

In the context of obesity-related disorders, such as MASH, rodents are commonly used to induce steatosis through excessive energy substrate intake through high-carbohydrate or high-fat diets (Farrell et al, 2019; Soret et al, 2020). In addition, specific strains of mice with appetite defects, such as *ob/ob* (i.e., leptin deficient) or *db/db* (i.e., leptin receptor mutated) mice, exhibit overeating tendencies and can develop steatosis (Soret et al, 2020). Hepatic lipid sequestration can also be promoted through choline-deficient diets (Soret et al, 2020). The resulting excessive nutrition leads to central obesity and metabolic syndrome, characterized by glucose intolerance, insulin resistance (accompanied by hyperinsulinemia), which serve as prerequisites for the development of MASH. However, finding a comprehensive preclinical model that accurately transitions into MASH and replicates the full range of phenotypic features observed in humans, including metabolic, inflammatory, fibrotic traits, and hepatocellular carcinoma, remains a significant challenge.

To address the lack of a comprehensive model, certain minimum requirements should be followed. It is advisable to use at least two models that cover the entire spectrum of the disease, such as a high-sugar and high-fat defined model, along with a model based on choline deficiency. These models should be evaluated by an experienced pathologist who can assess various scores, such as NAFLD Activity Score (NAS) or other relevant scoring system (Alberti et al, 2009; Kleiner et al, 2005). This evaluation allows for a comprehensive assessment of key features like steatosis, inflammation, and fibrosis, capturing the multifaced nature of MASH pathology (Farrell et al, 2019). Incorporating multiple models and expert evaluation allows researchers to gain a more holistic understanding of MASH and improve the translational relevance of their findings.

Similar issues arise from the use of rodents to model progressive stages of ALD. No single rodent model completely recapitulates the progression of human ALD (Arteel, 2010; Lamas-Paz et al, 2018). While long-term feeding of mice and rats with ethanol-containing diets results in the development of early stages of ALD, current models do not replicate the non-resolving inflammation characteristic of severe AH. Studies combining acute on chronic exposure and/or combinations of insults, e.g., ethanol and LPS, are currently under investigation. Of particular importance is the failure of any of these chronic ethanol exposure models to generate fibrotic injury (Lee and Seki, 2023).

Finally, there is a growing appreciation amongst hepatologists that the etiology of metabolic liver disease in many patients results from the interaction of high fat diets/obesity combined with alcohol consumption (Farrell et al, 2019; Rinella et al, 2023). The Delphi multi-center consensus group proposes a new classification for patients with both metabolic and alcohol-related injury. This category is termed MET-ALD combining the criteria for metabolic dysfunction-associated steatotic liver disease (MASLD), equivalent to previous NAFLD, with additional heavy alcohol consumption (Rinella et al, 2023). Therefore, there is a pressing need to develop models that specifically model the interactions between obesogenic diets that model MASLD/NAFLD and ethanol exposure (MET-ALD) (Buyco et al, 2021).

Another recommendation, which also applies to preclinical drug trials, is to test the molecules on both male and female mice and employ preventive and therapeutic approaches. In therapeutic approaches, the disease should be induced, and then attempts made to reverse it. Clear criteria, similar to clinical trials in humans, need to be defined before experimentation to establish specific targets of intervention. Historically, there has been a preference for using male mice over female mice in research studies (Beery, 2018). One of the reasons behind this preference is the concern that the hormonal cycle in female mice can lead to behavioral variations, potentially affecting the accuracy and reliability of experimental results (Beery, 2018). However, this biased approach has created a significant imbalance in the representation of genders in research, which could contribute to the high failure rate of molecules during phase 2b of clinical trials (Ratziu and Friedman, 2023). By overlooking the importance of studying both male and female subjects, researchers may have missed crucial insights into the potential sex-specific differences in drug responses, ultimately hindering the progress of clinical development.

Methodology should be meticulously reported to enhance reproducibility across different laboratories and minimize technological biases. Factors such as animal housing conditions (including health status of the animal facility) and the composition of the microbiota can significantly influence disease development. In addition, the genetic background of mouse strains may confer obesity resistance, which should be considered during model selection (Ferrannini et al, 2016). To ensure rigorous and transparent reporting of animal research in metabolic studies, we strongly recommend following the ARRIVE guidelines established by major biomedical journal editors (Omary et al, 2016). These guidelines provide a framework for transparent and comprehensive reporting, documenting important methodological details to enhance reproducibility and reliability of experimental results in the field of metabolic diseases.

### Future directions and therapeutic implications

#### Potential therapeutic targets in the necroptotic pathway and current clinical trials

The extensive cellular and pre-clinical investigations reviewed here clearly indicate that targeting of the mediators of necroptosis may be of clinical importance in prevention and treatment of metabolic liver diseases. However, given the complex cell and disease state functions of RIP1, RIP3, and MLKL, safe and effective therapeutic targeting poses many challenges. To date, the development of inhibitors of RIPK1 for clinical use is the most well advanced, with at least 5 clinical trials in process or terminated for indications ranging from ulcerative colitis to auto immune disease (Gardner et al, 2023; Mifflin et al, 2020). Compounds targeting RIPK3 activity are not as well developed. GlaxoSmithKline developed several RIPK3 inhibitors (GSK’840, GSK’843, GSK’872), but found they could trigger apoptosis at higher concentrations (Gardner et al, 2023; Mandal et al, 2014b). While several FDA approved drugs have off target specificity for inhibition of RIPK3 and some alternative small molecules are in pre-clinical development, no RIPK3 inhibitors are currently in clinical trials, to the best of our knowledge (Xia et al, 2020a). Necrosulfonamide is the most common inhibitor of MLKL and additional small molecule inhibitors of MLKL are in development, either targeting the pseudo-kinase domain or oligomerization (Gardner et al, 2023); however, their development also lags behind that of RIPK1 inhibitors.

Since many of the small molecule inhibitors in the necroptotic pathway have resulted in activation of other cell death pathways, some investigators have suggested that targeting necroptosis response and/or interacting pathways, rather than directly targeting the mediators of necroptosis, may be a more fruitful strategy (Vucur et al, 2023; Zhang et al, 2023b). For example, Vucur and colleagues demonstrated that inhibition of NF-кB limited RIPK3-mediated immune responses in a model of liver cancer and prevented cancer progression (Vucur et al, 2023). STING inhibitors have also been suggested as a potential mechanism to target necroptotic signaling in models of sepsis, due to the interactions between these two signaling pathways (Zhang et al, 2023b).

#### Considerations for future research directions: basic mechanisms to clinical applications

There are a number of unanswered questions as to the function of necroptotic signaling pathway in both in maintaining a healthy liver and their role in metabolic disease progression. Future studies aimed to clarify the non-canonical functions of RIP1, RIP3, and MLKL in multiple cells and tissues, including hepatocytes, immune cells, adipocytes, and intestinal epithelial cells will be important for integrating their function in multiple metabolic pathways. Particularly fascinating is the role that RIP1-RIP3-MLKL play in both regulation of homeostatic metabolic functions, including adipocyte differentiation (Magusto et al, 2022), insulin signaling, lipogenesis, mitochondrial biogenesis, and autophagic flux, compared to their role in cell death (Majdi et al, 2020; Saeed et al, 2019; Wu et al, 2020b; Xu et al, 2019). Understanding how these functions relate to the canonical pathway of necroptosis will likely aid in the development of very targeted and specific therapeutics. Further understanding on alternative pathways for MLKL activation, independent of RIP3, are also critical to clarify the role of MLKL in its multiple intracellular activities, including regulation of phagocytosis (Wu et al, 2023), autophagy (Wu et al, 2020b) and EV synthesis (Martens et al, 2021). Finally, given the differential contributions of RIP3 and MLKL to different murine models of metabolic liver disease (Gautheron et al, 2014; Miyata et al, 2022; Roychowdhury et al, 2013), further investigations may likely reveal distinct pathogenic mechanisms between ALD and MASLD/MASH and develop potential biomarkers to help improve diagnosis of patients with different etiologies of metabolic liver disease.

## Conclusion

In this review, we have summarized the progress made over the last decade in understanding the complex contributions of RIP1, RIP3, and MLKL to metabolic liver disease. The progression of metabolic liver diseases is complex, involving multiple cell-cell and organ-organ interactions. Accumulating evidence implicates necroptotic signaling in multiple cells and organs contributing to metabolic liver diseases, including immune cells, hepatocytes, adipocytes, and intestinal epithelium. While the necroptotic signaling pathway is an enticing therapeutic target, multiple challenges remain before this pathway can be effectively and safely regulated. Future studies will be required to fully understand the canonical and multiple non-canonical functions of RIP1, RIP3, and MLKL. It is very likely that cell-specific targeting of therapeutics may be necessary, given the contrasting functions of necroptotic signaling in different cells and organs. Furthermore, it will be important to understand the interplay between necroptotic signaling and other signaling pathways critical to maintaining metabolic, immune and liver homeostasis, including NF-κB and the STING pathways. Despite these challenges, investigators are continuing to advance our understanding of the multiplicity of functions that the proteins in the necroptotic signaling pathway contribute to maintaining health and their contribution to metabolic liver diseases.

### Pending issues

1. Investigate hepatocyte susceptibility to necroptosis considering RIP3 variable expression among different cell types and potential modulation by promoter methylation.
2. Understand the potential role of necroptosis mediators in regulating gene expression in the nucleus.
3. Elucidate how mediators of necroptosis orchestrate and regulate many forms of programmed cell death.
4. Develop specific conditional knockout mouse models to better define the role of necroptosis mediators in metabolism.
5. Initiate the testing of necroptosis inhibitors in the context of metabolic diseases to explore their potential as therapeutic targets.

## Glossary

Alcohol-associated liver disease (ALD): ALD is a spectrum of liver disorders and damage caused by excessive and prolonged alcohol consumption. ALD typically develops over time due to the toxic effects of alcohol on the liver, which can result in inflammation, fatty deposits, and scarring of the liver tissue.
Apoptosis: Apoptosis is a highly regulated process responsible for the elimination of unnecessary or damaged cells. This cellular mechanism plays a crucial role in shaping tissue or organ structure during embryonic development or larval metamorphosis in insects. Importantly, apoptosis can also occur at any time in adult cells when tissues need remodeling. The initiation of apoptosis can be triggered by signals originating from within the cell or from external factors that activate specific receptors on the cell’s outer membrane. Enzymes called caspases are responsible for orchestrating the controlled dismantling of the cell, ensuring that it undergoes self-destruction without causing harm to surrounding tissue.
Diabetes: A complex disorder of carbohydrates, fat, and protein metabolism primarily resulting from a relative or complete lack of insulin secretion by the beta cells of the pancreas or from defects of the insulin receptors. Diabetes affects the body’s ability to regulate blood sugar levels, leading to elevated glucose concentrations in the bloodstream. It is associated with various health complications and requires careful management through dietary control, medication, and, in some cases, insulin therapy.
Metabolism: It encompasses all chemical reactions within an organism that sustain life. It is commonly categorized into catabolic and anabolic processes. Catabolism involves breaking down complex substances into simpler forms, releasing energy, while anabolism uses this energy to construct cellular components like proteins and nucleic acids.
Mixed lineage kinase domain-like protein (MLKL): MLKL serves as the executioner in the process of necroptosis, triggering the disruption of the plasma membrane, which is a defining feature of this particular mode of programmed cell death.
Necroptosis: Necroptosis is a form of programmed cell death that distinguishes itself from apoptosis, a well-known mechanism of cell death dependent on caspases. It can be triggered by a combination of death ligands and death receptors under the inhibition of caspases, offering an alternative pathway for programmed cell death. It hinges on the formation of the necrosome complex, comprising receptor-interacting protein kinases (RIPKs) and Mixed lineage kinase domain-like protein (MLKL). Necroptosis shares morphological features with necrosis, including the early disruption of membrane integrity, cell and intracellular organelle swelling.
Non-alcoholic fatty liver disease (NAFLD)/metabolic dysfunction-associated steatotic liver disease (MASLD): NAFLD is a medical condition characterized by the accumulation of fat in the liver, known as hepatic steatosis. It is diagnosed when fat is found in the liver through imaging of liver histology, and other potential causes of liver fat accumulation, such as excessive alcohol consumption or certain medications, have been ruled out. NAFLD is associated with metabolic factors like obesity, insulin resistance, and high cholesterol lipids and is now replaced by the term MASLD due to its strong connection with these metabolic issues.
Non-alcoholic steatohepatitis (NASH)/metabolic associated steatohepatitis (MASH): NASH is a progressive form of NAFLD characterized by inflammation and liver cell damage in addition to the accumulation of fat in the liver. Unlike simple fatty liver, NASH is considered more severe and can lead to complications such as cirrhosis and liver cancer. The terminology for this condition is evolving, and it is now referred to as MASH to better reflect its connection with metabolic factors.
Obesity: It is a medical condition characterized by an excessive and unhealthy accumulation of body fat. It is typically determined by calculating the body mass index (BMI), where an individual’s weight is divided by the square of their height. A BMI of 30 or higher is generally considered indicative of obesity. It is often the result of a combination of genetic, environmental, and lifestyle factors, such as poor diet and lack of physical activity.
Receptor-interacting protein kinases (RIPKs): RIPKs are a group of highly conserved serine/threonine kinases throughout the course of evolution. These proteins are integral components of the cell’s signaling machinery, assembling multi-molecular complexes that have far-reaching implications, including the induction of apoptosis, necroptosis, inflammasome activation, and the activation of nuclear factor kappa B (NF-κB).
