# Author Correction: Mediators of necroptosis: from cell death to metabolic regulation

**DOI:** 10.1038/s44321-024-00095-1

**Published:** 2024-06-18

**Authors:** Xiaoqin Wu, Laura E Nagy, Jérémie Gautheron

**Affiliations:** 1 Northern Ohio Alcohol Center, Department of Inflammation and Immunity, Cleveland Clinic, Cleveland, OH USA; 2 Department of Integrative Biology and Pharmacology, McGovern Medical School, University of Texas Health Science Center at Houston, Houston, TX USA; 3 Department of Gastroenterology and Hepatology, Cleveland Clinic, Cleveland, OH USA; 4 Department of Molecular Medicine, Case Western Reserve University, Cleveland, OH USA; 5 Sorbonne Université, Inserm UMRS_938, Centre de Recherche Saint-Antoine (CRSA), Paris, 75012 France

## Abstract

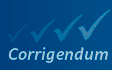

**Correction to:** *EMBO Molecular Medicine* (2024) 16:219–237. 10.1038/s44321-023-00011-z | Published online 9 January 2024

**The Acknowledgment section is corrected**.

JG is funded by the Agence Nationale de la Recherche (ANR-21-CE18-0002-01)

Is corrected to: (Changes in bold.)

JG is funded by the Agence Nationale de la Recherche (**ANR-21-CE17-0002**)

